# The Roles of Intracellular Chaperone Proteins, Sigma Receptors, in Parkinson’s Disease (PD) and Major Depressive Disorder (MDD)

**DOI:** 10.3389/fphar.2019.00528

**Published:** 2019-05-21

**Authors:** Kai Yang, Changcai Wang, Taolei Sun

**Affiliations:** ^1^ School of Chemistry, Chemical Engineering and Life Science, Wuhan University of Technology, Wuhan, China; ^2^ State Key Laboratory of Advanced Technology for Materials Synthesis and Processing, Wuhan University of Technology, Wuhan, China

**Keywords:** Sigma-1 receptor, PD, MDD, oligomerization, Sigma-2 receptor

## Abstract

Sigma receptors, including Sigma-1 receptors and Sigma-2 receptors, are highly expressed in the CNS. They are intracellular chaperone proteins. Sigma-1 receptors localize mainly at the mitochondria-associated endoplasmic reticulum (ER) membrane (MAM). Upon stimulation, they translocate from MAM to plasma membrane (PM) and nucleus, where they interact with many proteins and ion channels. Sigma-1 receptor could interact with itself to form oligomers, its oligomerization states affect its ability to interact with client proteins including ion channels and BiP. Sigma-1 receptor shows high affinity for many unrelated and structurally diverse ligands, but the mechanism for this diverse drug receptor interaction remains unknown. Sigma-1 receptors also directly bind many proteins including G protein-coupled receptors (GPCRs) and ion channels. In recent years, significant progress has been made in our understanding of roles of the Sigma-1 receptors in normal and pathological conditions, but more studies are still required for the Sigma-2 receptors. The physiological roles of Sigma-1 receptors in the CNS are discussed. They can modulate the activity of many ion channels including voltage-dependent ion channels including Ca^2+^, Na^+^, K^+^ channels and NMDAR, thus affecting neuronal excitability and synaptic activity. They are also involved in synaptic plasticity and learning and memory. Moreover, the activation of Sigma receptors protects neurons from death *via* the modulation of ER stress, neuroinflammation, and Ca^2+^ homeostasis. Evidences about the involvement of Sigma-1 receptors in Parkinson’s disease (PD) and Major Depressive Disorder (MDD) are also presented, indicating Sigma-1 receptors might be promising targets for pharmacologically treating PD and MDD.

## The Introduction of Sigma Receptors

### Sigma Receptors

Sigma receptors were mistakenly categorized as one kind of the opioid receptors because they had high-affinity binding sites for SKF-10047, which is a classic opioid receptor ligand (Kourrich et al., 2012; Su et al., 2016). Later, researchers realized that they were totally different receptors (Kourrich et al., 2012; Su et al., 2016). Sigma receptors are abundant in the central nervous system (CNS); they are expressed in the hippocampus, cerebellum, basal ganglia, and spinal cord (Bouchard and Quirion, 1997; Seth et al., 2001). Two Sigma receptor subtypes, Sigma-1 receptor and Sigma-2 receptor, have been identified according to their different binding profiles and molecule weights (Penke et al., 2018). While the Sigma-1 receptor was cloned many years ago, the identity of Sigma-2 receptor still remains uncertain, although several candidates have been proposed (Rousseaux and Greene, 2016). In recent years, significant progress has been made in our understanding of roles of the Sigma-1 receptors in normal and pathological conditions, but more studies are still required for the Sigma-2 receptor due to the lack of suitable research tools (Kourrich et al., 2012; Su et al., 2016). It is well accepted that Sigma receptors are non-G protein-coupled receptors (non-GPCRs); they are intracellular chaperone proteins (Penke et al., 2018). They can bind many unrelated and structurally diverse ligands and are involved in many cellular functions. Sigma-1 receptor plays very important roles in many CNS diseases such as Major Depressive Disorder (MDD), Parkinson’s disease (PD), and Alzheimer’s disease (AD) (Penke et al., 2018). Whereas Sigma-2 receptor is involved in cancers and AD, Sigma-2 receptor antagonists block the memory impairments in AD (Abate et al., 2018).

Sigma receptors localize mainly at the mitochondria-associated endoplasmic reticulum (ER) membrane (MAM) (Hayashi and Su, 2007) and in some plasma membranes (PM) (Mavlyutov and Ruoho, 2007; Mavlyutov et al., 2010). Upon stimulation, Sigma receptors translocate from the MAM to the PM, where Sigma receptors interact with many receptors and ion channels (Su et al., 2016). Sigma receptor might also move from the MAM to the nucleus membrane to interact with nucleus factors and regulate gene transcription (Tsai et al., 2015).

#### Sigma-1 Receptor

The Sigma-1 receptor shares no amino acid sequence homology with any other mammalian proteins. However, it has 60% amino acid identity to an enzyme, ERG2, which is required for sterol synthesis in yeast, whereas the Sigma-1 receptor does not have any enzyme activity (Kourrich et al., 2012; Su et al., 2016). The interaction between Sigma-1 receptor and sterol has been well studied, and several sterols and lipids could act as endogenous ligands for Sigma-1 receptor. For these interactions, tyrosine residues in Sigma-1 receptors are required (Palmer et al., 2007).

Hydrophobicity analysis has indicated that Sigma-1 receptor has two transmembrane domains, including amino acid (AA) sequence 11–29 and 92–112, which are named the transmembrane domain I (TMDI) and transmembrane domain II (TMDII), respectively. It is proposed that AA 29–92 forms a cytosolic loop and both the C- and N-termini are localized in the ER lumen (Su et al., 2016). In contrast to this two-pass transmembrane model, a recent study has revealed that Sigma-1 receptor has a single transmembrane topology, AA 6–31 comprises the single transmembrane helix, while AA 32–223 forms a C-terminal domain which is located in the cytosol. This domain includes a β-barrel and two flanking α-helices. Both the ligand-binding site and the oligomerization interface lie in this domain (Schmidt et al., 2016).

#### Sigma-2 Receptor

Up now, the molecular identity of Sigma-2 receptor has long been pursued. In 2006, Colabufo et al. proposed that Sigma-2 receptors are histone proteins since specific Sigma-2 ligand PB28 could pull down histone proteins (Colabufo et al., 2006). However, later studies discarded this conclusion.

Sigma-2 receptor is also suggested as a component of the progesterone receptor membrane component 1 (PGRMC1) complex (Xu et al., 2011). It is involved in cholesterol synthesis. Later, many articles were published to support this conclusion (Izzo et al., 2014a,b; Yi et al., 2017). However, this hypothesis had some serious drawbacks. For instance, the molecular size of PGRMC1 is different from that of the Sigma-2 receptor (Hiranita, 2016). In addition, the Sigma-2 receptor could still be photoaffinity-labeled in the cells without PGRMC1 (Chu et al., 2015; Abate et al., 2018).

Recently, transmembrane protein 97 (TMEM97) was thought to have the same identity as the Sigma-2 receptor. TMEM97 is located in the ER and has the ability to regulate the sterol transporter Niemann-Pick disease, type C1 Protein (NPC1) (Alon et al., 2017b). In that study, the authors found that its pharmacologic profile is the same as that of Sigma-2 receptor; moreover, TMEM97 ligands bind Sigma-2 receptors (Alon et al., 2017a). A new study has also shown that Sigma-2 receptor/TMEM97 is involved in alcohol withdrawal behaviors, indicating that this receptor can be targeted to treat alcohol use disorder (Scott et al., 2018).

These inconsistent studies leave the identity of Sigma-2 receptor open to debate. Over the years, many papers have shown that Sigma-2 receptor might be a promising target for AD and cancer. However, its unknown molecular identity has hindered further study. In addition, we still don’t know its 3D structure. Although Sigma-2 receptor shares some pharmacological properties with Sigma-1 receptor, these two proteins have different roles in the CNS. This review will focus on Sigma-1 receptor.

#### The Oligomerization of Sigma Receptor

Up to now, all the studies about oligomerization of Sigma receptors lie on Sigma-1. It is well known that Sigma-1 receptor could interact with itself and form oligomers (Gromek et al., 2014; Mishra et al., 2015). In one study, several high-molecular weight bands representing oligomers of Sigma-1 receptor appeared in rat liver membrane (Pal et al., 2007). A broad range of oligomerization states of Sigma-1 receptor was also revealed using SEC-MALS (size-exclusion chromatography with multi-angle light scattering) and PAGE (native polyacrylamide gel electrophoresis) (Schmidt et al., 2016). In addition, X-ray crystallization showed that human Sigma-1 receptor existed as a trimer with a transmembrane domain and a ligand binding site in each protomer (Schmidt et al., 2016). A GXXXG motif of the Sigma-1 receptor is required for its oligomerization, since mutations within this region reduced its higher states of oligomerization while increased smaller oligomeric states (Gromek et al., 2014). These mutations also prevented ligand binding (Gromek et al., 2014).

**Figure 1:**
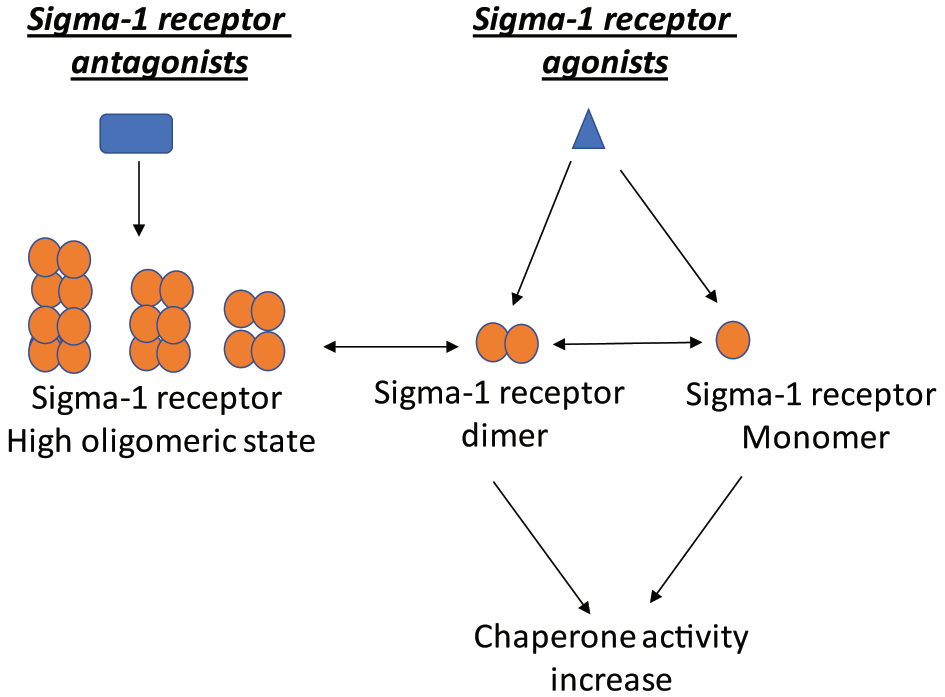
Sigma-1 receptor could form a range of different oligomeric states. While antagonist stabilized higher order oligomers, agonist instead favored small oligomers.

Since Sigma-1 receptor could form a range of different oligomeric states, it is not surprising that different agonists and antagonists of Sigma-1 receptor induce distinct cellular effects through its distinct oligomeric states (Mishra et al., 2015; Yano et al., 2018). A cell-based study using fluorescence resonance energy transfer (FRET) method showed that under non-liganded condition, Sigma-1 receptors form several different oligomeric states. When Sigma-1 receptors bind to their agonists or antagonists, antagonist favored higher order oligomer of receptors, while agonist enhanced the formation of small oligomers (Figure 1; Mishra et al., 2015). Consistently, in a non-denaturing gel assay, Sigma-1 receptor antagonist haloperidol promoted higher order oligomerization while agonist pentazocine favored its monomer and dimer states (Yano et al., 2018).

The promising hypothesis is that Sigma-1 receptor oligomerization states might affect its ability to bind client proteins including ion channels and BiP. For example, the preincubation of Sigma-1 receptor with ligands reduced the binding of Sigma-1 receptor monomer with the Nav 1.5 channel (Balasuriya et al., 2012). It is proposed that Sigma-1 receptor ligands including haloperidol and pentazocine could stabilize higher oligomeric (includes dimeric) states of the receptors, so less receptor monomers are available for the binding of Nav 1.5 channel. In addition, the conversion of oligomeric states of the receptor to its monomer might enhance its binding ability to client protein including BiP, since more C-terminus of the receptor was exposed in monomer. A recent correlation experiment showed that haloperidol which promoted the homomerization of the Sigma-1 receptor reduced Sigma-1 receptor-BiP interaction, whereas pentazocine which favored the production of monomers enhanced the interaction between Sigma-1 receptor and BiP (Yano et al., 2018).

### Signal Pathways Induced by the Activation of Sigma Receptors

The activation of Sigma-1 receptors could induce several signaling pathways in cells (Su et al., 2016). It modulates Ca^2+^ signaling *via* the inositol triphosphate (IP_3_) receptor between the ER and mitochondria, and this regulation ensures the Ca^2+^ signaling from ER into mitochondria (Hayashi and Su, 2007). In addition, another Ca^2+^ channel, Ryanodine receptor (RyR), is also activated by Sigma-1 receptor agonists (Tagashira et al., 2013).

Sigma-1 receptor is involved in the generation of reactive oxygen species (ROS). Its activation inhibits ROS generation in cells. ROS produced by NADPH oxidase is increased in neurons with reduced Sigma-1 receptor expression (Pal et al., 2012). Further, ROS level is elevated in retinal Müller cells deficient in Sigma-1 receptor (Wang et al., 2015). ROS production induced by lipopolysaccharide (LPS) was also reduced by Sigma-1 receptor agonist, (+)-pentazocine, in retinal microglia (Zhao et al., 2014). In contrast, in the spinal cord and brain mitochondria, Sigma-1 receptor agonists increase the production of ROS (Choi et al., 2013; Natsvlishvili et al., 2015). These conflicting results regarding the effect of Sigma-1 agonists and antagonists on the ROS generation require further study.

It is proposed that the activation of Sigma-1 receptor modulates the antioxidant response elements; therefore, its agonists could be used as indirect antioxidants (Pal et al., 2012). Inositol-requiring enzyme 1 (IRE1) is one of the ER stress sensors residing specifically at the MAM. Under ER stress, the Sigma-1 receptor was dissociated from BiP and its chaperone activity was activated, which stabilized IRE1 at the MAM, then the dimerization of IRE1 resulted in its activation. Once activated, IRE1 upregulated the transcription of several ER chaperones to counteract the ROS-induced stress on cells (Mori et al., 2013; Hayashi, 2015). Sigma-1 receptors also attenuate the production of ROS by enhancing the nuclear factor (erythroid-derived 2)-like 2 (Nrf2) signaling pathway (Wang et al., 2015).

### Sigma Receptor Interactions With Proteins

The Sigma-1 receptor is able to bind many proteins including ion channels such as inositol triphosphate (IP_3_) receptors (Hayashi and Su, 2007), voltage-gated K^+^ (Kourrich et al., 2013), Na^+^ (Johannessen et al., 2009), Ca^2+^ channels (Tchedre et al., 2008), and NMDA receptors (Pabba and Sibille, 2015). In addition, the Sigma-1 receptor could directly modulate neuronal mitochondrial Rac-1 GTPase activity (Natsvlishvili et al., 2015).

Binding immunoglobulin protein (BiP), also known as 78-kDa glucose-regulated protein (GRP78) or heat shock 70-kDa protein 5 (HSP5), is a protein located in the MAM as a molecular chaperone. Sigma-1 receptor interacts with BiP which is Ca^2+^-sensitive under physiological conditions (Hayashi and Su, 2007; Ortega-Roldan et al., 2013). However, the decrease of the local Ca^2+^ concentration, or Sigma-1 receptor agonists, causes Sigma-1 receptor to dissociate from BiP, resulting in its redistribution from MAM to the ER and the increase of its chaperone activity (Hayashi and Su, 2007).

Sigma-1 receptors also interact with many GPCRs including opioid receptor, corticotropin-releasing factor (CRF) receptor, orexin receptor, and dopamine receptors (Kim et al., 2010; Navarro et al., 2010, 2013, 2015, 2018; Beggiato et al., 2017; Feltmann et al., 2018). Although Sigma-1 receptor doesn’t belong to the opioid receptor family, it physically associates with mu opioid receptor. This association was demonstrated by Co-IP experiments using epitope-tagged receptors in HEK293 cells. Sigma-1 receptors could modulate opioid function without changing its binding (Kim et al., 2010).

In addition, bioluminescent resonance energy transfer-based technique (BRET) experiments showed that a direct interaction between Sigma-1 receptor and CRF1 receptor exists in HEK293 cells. CRF1 receptor participates in cocaine-dependent actions *via* Sigma-1 receptor – CRF1 receptor oligomer (Navarro et al., 2015). Similarly, Sigma-1 receptor also binds CRF2 receptor physically, which mediates amphetamine effects on CRF2 receptor (Navarro et al., 2018).

Interestingly, although Orexin1 receptor is not able to physically interact with Sigma-1 receptor (Navarro et al., 2015), there is a direct interaction between Orexin-1 receptor and Sigma-2 receptor demonstrated by BRET assay, amphetamine affects Orexin1 receptor function *via* this binding (Navarro et al., 2018).

Sigma-1 receptor also binds dopamine receptor. Dopamine receptors consist of at least five subtypes (D1R, D2R, D3R, D4R, and D5R); D1-like receptors include D1R and D5R which stimulate adenylyl cyclase, while D2-like receptors include D2R, D3R, and D4R which inhibit adenylyl cyclase (Beaulieu and Gainetdinov, 2011). Using BRET, a direct interaction between Sigma-1 receptor and dopamine D1 receptor was reported in murine striatal slices (Navarro et al., 2010). A similar method was used to study the functional interaction between Sigma-1 receptor and dopamine D2-like receptor. It has demonstrated that only D2 receptor could physically interact with Sigma-1 receptors while D3 and D4 receptors did not in mouse striatum. Cocaine inhibited downstream signaling in both cultured cells and mouse striatum by interacting with Sigma-1 receptor – D2 receptor complex (Navarro et al., 2013). This complex also exists in rat striatal dopaminergic and glutamatergic terminals (Beggiato et al., 2017). In addition, the expression of this complex was reduced in rat alcohol drinking model (Feltmann et al., 2018).

### Sigma Receptor Ligands

The Sigma-1 receptor shows high affinity for many unrelated and structurally diverse ligands (Table 1). They include cocaine (Kourrich et al., 2013), methamphetamine (Hedges et al., 2018), the antipsychotic drug haloperidol (Yano et al., 2018), and antidepressants such as fluoxetine (Safrany and Brimson, 2016). The actions of these drugs could be mediated by the Sigma-1 receptor.

**Table 1:** The list of Sigma-1 receptor agonists and antagonists.

Sigma receptor ligands – agonists	Sigma receptor ligands – antagonists
PD 144418	BD 1063
4-PPBP	BD 1047
Pentazocine	NE-100
(+)-SKF 10,047	BMY 14802
PRE-084	Haloperidol
Pregnenolone sulfate (PREGS)	
Dehydroepiandrosterone sulfate (DHEAS)	
SA 4503	
Fluvoxamine	
DTG	
SKF 83959	
MDMA	
Memantine	
Phencyclidine	
Heroin	
Cocaine	
Methamphetamine	
Dehydroepiandrosterone (DHES)	
SOMCL-668	
LS-1-137	

In order to explain this diversity, it was suggested that the Sigma-1 receptors possess flexible structures, which could bind all these structurally diverse compounds. In 2005, Glennon et al. proposed a model for pharmacophore for the Sigma-1 receptor ligands (Glennon et al., 1994). This pharmacophore includes a central basic amine nitrogen atom surrounded by two hydrophobic features. One feature is a primary hydrophobic group which is 6–10 Å away from the central nitrogen atom. The other one is a secondary hydrophobic group that is located 2.5–3.9 Å from the nitrogen atom. This feature could interact with the bulky substituent of ligands without affecting their binding affinity (Glennon et al., 1994).

Up to now, because of lack of a clear-cut methodology, we still don’t understand the mechanism of this diverse ligand-receptor interaction. Recently, a new method was developed to address this dilemma. In this study, a Sigma-1 receptor biosensor consisting of CFP and YFP was constructed; when ligand bound to Sigma-1 receptor, it induced the receptor to rearrange its intramolecular conformation, which was monitored by real-time FRET-based assay. By this way, we could compare the intrinsic activity of different Sigma-1 receptor ligands. It has shown that Sigma-1 receptor agonists or antagonists induced conformation of Sigma-1 receptors to be rearranged in an opposite manner (Gomez-Soler et al., 2014).

## The Physiological Roles of Sigma Receptor in the Central Nervous System

### Neuronal Excitability

Through both indirect and direct physical interactions, the Sigma-1 receptor could modulate various ion channels including voltage-gated Ca^2+^, Na^+^, and K^+^ channels, which involve both action potential’s generation and its conduction; by this way, the Sigma-1 receptor has the ability to affect cell excitability of the neuron (Kourrich et al., 2012).

The Sigma-1 receptor could enhance or inhibit Ca^2+^ channel activity through either direct or indirect actions (Kourrich et al., 2012). To date, only few studies have shown the direct action of Sigma-1 receptor on Ca^2+^ channels in the CNS (Mueller et al., 2013; Zhang et al., 2017). In cultured retinal ganglion cells, the activation of Sigma-1 receptor by SKF10047 inhibited Ca^2+^ currents, and this effect was abolished by Sigma-1 receptor antagonist BD 1047. In addition, the association between the Sigma-1 receptor and Ca^2+^ channel was shown by Co-IP experiments (Mueller et al., 2013). N-type Ca^2+^ currents from cholinergic interneurons in rat striatum were also reduced by Sigma-1 receptor agonists (SKF-10047 and Pre-084) (Zhang et al., 2017), this inhibition was prevented by Sigma-1 receptor antagonist BD 1063. Sigma-1 receptors and N-type Ca^2+^ channels bind together, which is demonstrated by FRET assays and Co-IP (Zhang et al., 2017).

In contrast, Sigma-1 receptor activated by pregnenolone sulfate (PREGS) enhanced the activity of L-type Ca^2+^ channel and facilitated LTP induction in hippocampal slices, the involvement of Sigma-1 receptor demonstrated that Sigma-1 receptor antagonist BD 1047 prevented this PREGS induced facilitation. But, whether Sigma-1 receptor interacted with Ca^2+^ channels directly was not studied (Sabeti et al., 2007).

For Na^+^ channel, the Sigma-1 receptor could inhibit its activity both directly and indirectly (Cheng et al., 2008; Balasuriya et al., 2012). In prefrontal cortex slice, the Sigma-1 receptor activated by dehydroepiandrosterone sulfate (DHEAS) inhibits Na^+^ current through Gαi protein and protein kinase C (PKC) pathway (Cheng et al., 2008). However, several other studies have shown that the Sigma-1 receptor can also modulate Na^+^ current directly through physical interaction (Balasuriya et al., 2012). In cardiomyocytes, Sigma-1 receptor ligands reduce Nav1.5-dependent currents; this reduction of Na^+^ current doesn’t require the inclusion of ATP and GTP, indicating the direct action of Sigma-1 receptor on Na^+^ channels (Johannessen et al., 2009). Atomic force microscopy (AFM) studies have revealed that Sigma-1 receptor directly binds Na^+^ channels with a four-fold symmetry (Balasuriya et al., 2012).

The regulation of K^+^ channels by Sigma-1 receptor involves either indirect mechanisms or direct protein–protein interactions; it could modulate K^+^ channels’ function and trafficking (Su et al., 2010; Kourrich et al., 2012). In cortical neurons, the Sigma ligands increase the Kv2.1-dependent current *via* Gi/o-dependent pathway (Soriani et al., 1998). To date, evidences for a direct physical interaction between Sigma-1 receptor and K^+^ channels also appear. The activation of the Sigma-1 receptor could differentially inhibit the Kv1.4 current, which suggests that the Sigma-1 receptor might act as a K^+^ channel subunit (Aydar et al., 2002). It is reported that the interaction between Sigma-1 receptor and Kv channels also occurs in the brain (Kourrich et al., 2013). The activation of Sigma-1 receptor enhances the trafficking of Kv1.2 channels to the PM in both the nucleus accumbens (NAc) and the prefrontal cortex (PFC), which is proposed to be mediated by Sigma-1 receptor-Kv1.2 interaction. In addition, *in vivo* injection of cocaine increases Kv1.2 current in NAc neurons *via* this interaction (Kourrich et al., 2013).

The effects of the Sigma-1 receptor on overall neuronal activity are brain region- and cell type-specific. Usually, the inhibition of Na^+^ channels by the Sigma-1 receptor (Cheng et al., 2008; Balasuriya et al., 2012) decreases action potential, whereas inhibition of K^+^ channels (Aydar et al., 2002; Kourrich et al., 2013) increases it. Since Sigma-1 receptor activation has opposite effects on these voltage-dependent ion channels, it is impossible to infer its effects on the overall neuronal activity based on the combination of their diverse effects on these channels. Furthermore, ion channels involved in neuronal excitability have many subtypes, and Sigma-1 receptor might have opposite effects on these different subtypes, which make the role of Sigma-1 receptor on neuronal excitability more complicated. In conclusion, the overall effect of Sigma-1 receptor on neuronal activity may depend on both the expression level of Sigma-1 receptor and the subtypes of voltage-dependent ion channels.

### Synaptic Plasticity, Learning and Memory

A well-accepted cellular model of learning and memory in the hippocampus is synaptic plasticity, including LTP and LTD; both forms are NMDA receptor (NMDAR) dependent (Paoletti et al., 2013). So NMDAR is important for synaptic plasticity and learning and memory (Paoletti et al., 2013).

It is demonstrated that Sigma-1 receptor ligands have the ability to regulate NMDAR activity bidirectionally. Sigma-1 receptor agonists increase NMDAR activity in many brain regions, including the CA3 field of rat dorsal hippocampus (Sabeti et al., 2007; Balasuriya et al., 2013; Pabba et al., 2014). This potentiation of NMDAR by Sigma-1 receptor could be indirect through signaling pathways, and many studies have revealed that this enhancement maybe is mediated by inhibiting small-conductance K^+^-activated Ca^2+^ channels (SK channels) and/or through G proteins (Sabeti et al., 2007). In addition, Sigma-1 receptor could regulate NMDAR directly by physical interaction. Using AFM and *in situ* proximity ligation assays (PLA), Balasuriya et al. discovered a direct association between Sigma-1 receptor and NMDAR. Sigma-1 receptor bound directly to the GluN1 subunit of NMDAR (Balasuriya et al., 2013). Furthermore, the activation of Sigma-1 receptor enhanced the trafficking and surface expression of NMDARs in the hippocampus (Pabba et al., 2014).

Additionally, Sigma-1 receptor could function as a safety switch to control cannabinoid receptor 1 (CB1R)-NMDAR interactions, rescuing CB1R-induced NMDAR hypofunction (Rodriguez-Munoz et al., 2015). Sigma-1 receptors were found to form a complex consisting of CB1R, GluN1, and the histidine triad nucleotide-binding protein 1 (HINT1) (Rodriguez-Munoz et al., 2015). The activation of Sigma-1 receptor could restore the hypofunctional NMDAR induced by CB1R (Rodriguez-Munoz et al., 2015).

On the other hand, the activation of Sigma-1 receptor reduced NMDAR activity in rat cortical neurons (Liang and Wang, 1998), retinal ganglion neurons (Zhang et al., 2011), and midbrain dopaminergic neurons (Shimazu et al., 2000). The suppression of NMDAR function caused by the activation of Sigma-1 receptors is proposed to be mediated by a PLC-PKC pathway which is Ca^2+^-dependent (Zhang et al., 2011).

Although the modulation of NMDAR by Sigma-1 receptor has been well studied, the effect of Sigma receptor ligands on AMPAR is largely unknown. In rat retinal ganglion cells, Sigma-1 receptor ligands inhibit AMPAR activity; this inhibition is Ca^2+^ independent and PKG dependent, neither protein kinase A (PKA) nor phosphoinositide (PI)-phosphoinositide phospholipase C (PLC) pathways is involved (Liu et al., 2016).Since Sigma-1 receptor has the ability to modulate both NMDARs and AMPARs, it is not surprising that Sigma-1 receptor could affect synaptic plasticity. LTP was reduced in Sigma-1 receptor knockout mice (Snyder et al., 2016). Additionally, in the hippocampus, the activation of Sigma-1 receptor increased LTP *via* the inhibition of SK channels (Sabeti et al., 2007).

In addition to synaptic plasticity, a new hypothesis was proposed recently. It claimed that the dynamic balance of membrane receptor complexes at synapses is the molecular basis for learning and memory (Borroto-Escuela et al., 2015, 2018). In this model, during short-term memory, learning can induce the transient reorganization of homo- and hetero-receptor complexes (made up of different receptors and adapter proteins including Sigma-1 receptors) in the postsynaptic membrane. This receptor reorganization then releases soluble factors like adenosine, ATP, growth factors, and ECVs from the postsynaptic membrane, which will reach the presynaptic membrane, resulting in the formation of a novel pattern of transmitter release (Borroto-Escuela et al., 2015, 2018). Subsequently, the presynaptic hetero-receptor complexes are also reorganized to maintain this new pattern of transmitter release, which reflects the firing pattern to be learned by the complexes in the postsynaptic membrane (Borroto-Escuela et al., 2015, 2018). For long-term memory, some hetero-receptor complexes in the postsynaptic membrane are converted into some unique transcription factors, which induce the synthesis of novel adaptor and scaffolding proteins. These new synthesized proteins then link these hetero-receptor complexes together or to the cytoskeleton, resulting in the formation of much more stable complexes. By this way, long-term memory is created (Borroto-Escuela et al., 2015, 2018).

Many studies have demonstrated that Sigma-1 receptor is involved in learning and memory. For example, Sigma-1 receptor agonists enhance the learning and memory behaviors in mice (Zvejniece et al., 2014). Further, when memory was impaired by phencyclidine (PCP) in rat novel object recognition, it could be rescued by Sigma-1 receptor activated by pridopidine (Sahlholm et al., 2018). In another study, LS-1-137, a Sigma-1 receptor agonist, could partially reverse the learning deficits induced by scopolamine administration in learning and memory behavior tests; scopolamine is a muscarinic receptor antagonist (Malik et al., 2015). Additionally, Ayahuasca, a psychoactive plant brew used for therapeutic and spiritual purposes, could heal traumatic memories *via* Sigma-1 receptor (Inserra, 2018).

### Neuroprotection

#### Modulation of Endoplasmic Reticulum Stress

When unfolded or misfolded proteins within the ER lumen are accumulated, they would activate three major signaling pathways to restore protein folding homeostasis; this is called the unfolded protein response (UPR), and these three major signaling pathways include protein kinase RNA-like ER kinase (PERK), activating transcription factor 6 (ATF6), and inositol requiring enzyme 1 alpha (IRE1α) (Hetz and Mollereau, 2014; Hetz and Saxena, 2017). The activation of UPR decreases the production of newly synthesized proteins and induces the degradation of misfolded proteins, which reduces the overload of the ER lumen by proteins. However, if the stress signal is severe and/or prolonged, it will result in cell death (Hetz and Mollereau, 2014; Hetz and Saxena, 2017).

As chaperones, Sigma-1 receptors could degrade unfolded and/or misfolded proteins to reduce ER stress. Under normal conditions, Sigma-1 receptor interacts with another ER-chaperone BiP directly (Su et al., 2010). During ER stress Sigma-1 receptor dissociates from BiP and then binds and stabilizes IRE1a, which reliefs ER stress. In addition, ER stress could upregulate the expression of Sigma-1 receptor (Mitsuda et al., 2011). For example, the activation of Sigma-1 receptors by fluvoxamine increases Sigma-1 receptor expression, which inhibits the death of Neuro2A cells (Omi et al., 2014).

#### Modulation of Ca^2+^ Homeostasis and Glutamate Toxicity

The regulation of intracellular Ca^2+^ homeostasis is one of the major mechanisms by which Sigma-1 receptor ligands perform neuroprotection. High concentration of Ca^2+^ in the cytosol results in cell death. In addition to influx through NMDARs and voltage-dependent Ca^2+^ channels, Ca^2+^ can also be released from intracellular ER and mitochondria stores.

In the MAM, the interaction between Sigma-1 receptors and BiP is sensitive to Ca^2+^ change (Hayashi and Su, 2007); Ca^2+^ depletion causes the dissociation of this complex, resulting in the redistribution of the Sigma-1 receptor from MAM to the ER (Hayashi and Su, 2007). In addition, this interaction also stabilizes IP_3_R at the MAM, regulating Ca^2+^ influx into mitochondria and thus influencing ATP production *via* the activation of the Krebs’ cycle (Hayashi and Su, 2007).

In the CNS, glutamate is the major excitatory neurotransmitter which could trigger the opening of NMDAR. Many studies have shown that hypofunctional NMDAR is detrimental to the neurons. The enhancement of NMDAR function by the activation of Sigma-1 receptor is beneficial under most situations. On the other hand, overactivated NMDAR is also not good for the neurons. When a large amount of Ca^2+^ flows into the cytosol *via* NMDARs, it activates downstream cell death pathways, including the activation of calpains and proteases (Szydlowska and Tymianski, 2010). Interestingly, under certain conditions, activation of Sigma-1 receptors could inhibit NMDAR activity (Liang and Wang, 1998; Shimazu et al., 2000; Zhang et al., 2011). This reduction of NMDAR function by Sigma-1 receptors might involve a Ca^2+^-dependent PLC-PKC pathway (Zhang et al., 2011). In addition to influencing the function of NMDARs, Sigma-1 receptor ligands also modulate glutamate release, although the mechanisms are poorly understood. In rat cerebral cortex, the application of Sigma-1 agonists reduced Ca^2+^ entry through the inhibition of presynaptic voltage-dependent Ca^2+^ channels, resulting in decreased glutamate release from nerve terminals (Lu et al., 2012). In conclusion, through the modulation of glutamate release and NMDARs, Sigma-1 receptors may dampen the excitotoxic effect of glutamate.

Sigma-1 receptor ligands might also affect the interaction of NMDARs with other proteins to protect neurons from death. It is proposed that when there is excess Ca^2+^ influx through NMDAR, it activates nNOS within the NMDAR-PSD95-eNOS complex; thus, a large amount of NO is produced which contributes to neurotoxicity (Sattler et al., 1999). The activation of sigma-1 receptor could reduce the interactions between GluN2B and PSD95 as well as PSD95 and nNOS, protecting the neurons against cell death (Yang et al., 2010). In addition, Sigma-1 receptor agonists also stimulated the binding of HINT1 to GPCRs which enhanced GPCR-NMDAR interactions; by this way, the activation of Sigma-1 receptor could protect the neurons against NMDAR-induced neurotoxicity (Rodriguez-Munoz et al., 2015).

#### Modulation of Neuroinflammation

Microglia are the primary innate immune effector cells in the CNS (Hickman et al., 2018; Song and Colonna, 2018). Under normal conditions, microglia constantly sense the changes in their environment (Hammond et al., 2018). The ability of microglia to react to CNS damage underlies their importance in neurodegeneration (Hickman et al., 2018; Song and Colonna, 2018). As a result of disruptions in CNS homeostasis, brain injury or other perturbations, microglia are activated and release pro-inflammatory mediators including cytokines and ROS, which result in neuronal death (Hickman et al., 2018; Song and Colonna, 2018).

Similar to macrophages, microglia usually have two phenotypes: M1 and/or M2 phenotypes. M1 microglia are pro-inflammatory and their activation is harmful to the CNS, while M2 microglia are anti-inflammatory and protective (Hickman et al., 2018; Song and Colonna, 2018). Sigma-1 receptors, also expressed in microglia, might modulate microglial activation and dampen neuroinflammation (Jia et al., 2018). Sigma-1 receptors regulate several aspects of microglial activation. In rat microglial cultures, the Sigma-1 receptor agonist DTG reduces the ability of microglia to release TNF-α, IL-10, and NO in response to ATP, MCP-1, and LPS (Zhao et al., 2014). In addition, in murine microglial BV2 cells, the activation of the Sigma-1 receptor prevented M1 microglial activation induced by LPS and decreased the release of inflammatory cytokines (Wu et al., 2015).

In contrast, in animals with motor neuron disease, the application of PRE084 increased M2 microglial phenotypes (Peviani et al., 2014). Sigma ligands improved microglial cell survival during ischemia or Aβ exposure in primary microglia cultures (Behensky et al., 2013). In conclusion, the activation of Sigma receptors may modulate microglial activity by enhancing its anti-inflammatory M2 phenotype while reducing its inflammatory response (M1).

In addition to microglia, astrocyte is also involved in neuroinflammation. Depending on the properties of injuries, when astrocytes are activated, they could release gliotransmitters, pro-inflammatory cytokines, and neuroprotective factors such as BDNF. Sigma-1 receptor is highly expressed in astrocytes, indicating its ability to modulate the functions of astrocytes. In cultured astrocytes, haloperidol induced GM1 accumulation in the autophagosomes of astrocytes *via* the activation of Sigma-1 receptor (Kasahara et al., 2017). GM1 is a sialic acid-containing glycosphingolipid, which is highly abundant in the CNS; it is involved in neuroinflammation (Schneider et al., 2019). Furthermore, in a mouse model of motor neuron degeneration (MND), the activation of Sigma-1 receptor by PRE-084 decreased reactive astrocytosis (Peviani et al., 2014). In addition, using neuronal-glial mixed cultures, GFAP expression was enhanced in Sigma-1 knockout mice compared to that of WT (Weng et al., 2017).

## The Role of Sigma Receptor in Parkinson’s Disease and Major Depressive Disorder

### Parkinson’s Disease

Parkinson’s disease (PD) is a common neurodegenerative disorder demonstrated by the massive death of dopaminergic neurons in substantia nigra pars compacta (SNpc) and the existence of Lewy bodies, which consist of α-synuclein (Poewe et al., 2017). Its classical motor features include bradykinesia, rest tremor, and muscle rigidity (Poewe et al., 2017). PD also has some non-motor symptoms such as memory impairment, sleep disorders, depression, and autonomic dysfunction, which affect the quality of patients’ life (Charvin et al., 2018). The etiology of sporadic PD remains to be unraveled, but it involves genetic, lifestyle, and environmental factors (Poewe et al., 2017).

Many penetrant genes associated with PD have been identified including α-synuclein, DJ-1, LRRK-2 (which encodes leucine-rich repeat kinase 2), and GBA (which encodes glucocerebrosidase) (Poewe et al., 2017). Mutations of these genes can cause abnormal α-synuclein proteostasis, mitochondrial dysfunction, oxidative stress, neuroinflammation, and motor circuit pathophysiology (Poewe et al., 2017). Oxidative stress is a potential driver for PD progression. The accumulation of oxidative damage markers in the substantia nigra has been demonstrated in brain tissues from patients with PD (Jenner and Olanow, 1996). Oxidative stress may accelerate α-synuclein aggregation (Cristovao et al., 2012) and cell death (Guo et al., 2018). Sigma-1 receptor agonists were found to reduce oxidative stress *via* several signaling pathways (Pal et al., 2012; Wang et al., 2015), indicating the beneficial role of Sigma-1 receptor in PD.

In addition, the Sigma-1 receptor binds cocaine and methamphetamine and gets involved in their behavioral and cellular effects on animals (Kourrich et al., 2013; Hedges et al., 2018). One study has demonstrated that Sigma-1 receptors are involved in dopamine (DA)-induced cell death in CHO cells (Mori et al., 2012). In CHO cells deficient in Sigma-1 receptor, physiological concentration of DA induced cell death. It is proposed that nuclear factor κB (NF-κB) p105 is converted to p50 by DA, which reduces the transcription of Bcl-2 (Mori et al., 2012). Therefore, endogenous Sigma-1 receptors could tonically inhibit DA-induced NF-κB activation, which protects cell from death. Thus, Sigma-1 receptor ligands may represent new therapeutic targets for PD.

In early stage PD patients, the level of Sigma-1 receptors is downregulated (Mishina et al., 2005). In addition, Francardo et al. (2014) treated 6-hydroxydopamine (OHDA) model mice with the Sigma-1 receptor agonist PRE-084 daily; after 5 weeks, these PD model mice exhibited a significant behavioral recovery (Francardo et al., 2014). Hong et al. (2017) reported that in Sigma-1 KO mice, the death of dopaminergic neurons increased. This phenomenon was caused by the aggregation and phosphorylation of α-synuclein (Hong et al., 2017). Whereas in 2015, the same group reported inconsistent results using the 1-methyl-4phenyl-1,2,3,6-tetrahydropyridine (MPTP) model mice. The authors found that deficiency of Sigma-1 receptor could reduce MPTP-induced death of dopaminergic neurons, which is mediated by the suppression of NMDAR function and DA transporter expression (Hong et al., 2015). These contradictory observations can be reconciled because they used different animal models. While MPTP is a mitochondrial toxicant, it may directly impact mitochondrial functions.

Recently, PD circuit dysfunction has attracted more and more attention. It likely represents a new direction for the treatment of PD (McGregor and Nelson, 2019). The basal ganglia which control the movement of body are the major brain areas affected in PD. They consist of several nuclei including the caudate nucleus, the putamen, the substantia nigra, and the globus pallidus. These nuclei form an interconnecting network which processes the signals for initiation and termination of movement (McGregor and Nelson, 2019). The death of dopaminergic neurons in substantia nigra pars compacta could induce dysregulation of the motor circuits that project throughout the basal ganglia (McGregor and Nelson, 2019). Up to now, whether Sigma-1 receptor is involved in basal ganglia circuit function remains unknown.

### Major Depressive Disease (MDD)

Major depressive disorder (MDD) is a debilitating disease with depressed mood and poor cognitive function (Otte et al., 2016). It is caused by both heritability and environmental factors. Environmental factors, including physical or emotional abuse during early life, are strongly linked to MDD (Otte et al., 2016). MDD is linked to the dysfunctions of dopaminergic, serotonergic, and glutamatergic systems (Otte et al., 2016). For example, it is very common to use selective serotonin reuptake inhibitor (SSRI) to treat depression; this inhibitor can block serotonin reuptake in the synaptic cleft, thus increasing the concentration of serotonin (Fink and Gothert, 2007). In addition, early life stress could induce depression by changing serotonin levels in the brain (Kawahara et al., 1993). Some SSRIs also act on dopaminergic system to exert their antidepressant effects (Prinz et al., 2013). Studies have shown that low level of dopamine is linked to MDD (Belujon and Grace, 2017). Additionally, the application of dopamine agonists can rescue the symptoms of depression (Leentjens, 2011). Recently, it has been shown that abnormal function of glutamatergic system also leads to MDD (Murrough et al., 2017). A strong evidence which supported this hypothesis is that the NMDAR blocker ketamine has the ability to rescue the core symptoms of depression (Yang et al., 2018). Furthermore, postmortem studies showed that in MDD patients, the expression and function of NMDAR subunits were altered (Feyissa et al., 2009).

Up to now, the first generation of antidepressants, SSRIs, has not helped approximately a third of patients with MDD. In addition, it usually takes several weeks to months for SSRIs to partially relieve the symptoms of MDD (Murrough et al., 2017). Although the monoamine systems (including the serotonin and dopamine systems) have been focused by depression research, now more and more researchers agree that the other targets should be pursued (Murrough et al., 2017). Recently, Sigma receptors have emerged as a promising target.

Polymorphisms and association analyses have suggested that Sigma-1 receptor is involved in MDD. A genetic link between Sigma-1 receptor and MDD was discovered in a Japanese population (Kishi et al., 2010). In addition, Sigma-1 receptor knockout mouse demonstrated increased depressive-like phenotype (Sabino et al., 2009). Mounting pharmacological data have suggested that many Sigma-1 receptor agonists play very important antidepressant roles (Matsuno et al., 1996; Wang et al., 2007). One study has shown that Sigma receptor agonists reduce depression (Matsuno et al., 1996; Ukai et al., 1998; Wang et al., 2007). Moreover, the application of SOMCL-668, which could modulate Sigma-1 receptor activity, decreased depressive-like behaviors in mice (Wang et al., 2016). Furthermore, antidepressant actions of the neurosteroids DHEAS and pregnenolone sulfate (PS) appear to be mediated by Sigma-1 receptors (Reddy et al., 1998; Urani et al., 2001). Antidepressant actions of several other compounds, including fluvoxamine, were prevented by Sigma-1 receptor antagonist (Wang et al., 2007; Dhir and Kulkarni, 2008).

The mechanism by which Sigma-1 receptors modulate antidepressant-like behavior is not understood. It is proposed that the modulation of serotonergic transmission may be involved since Sigma-1 receptor knockout mice with the increased depressive-like behavior also showed deficits in the activity of serotonergic neurons (Sabino et al., 2009). Electrophysiological studies demonstrated that Sigma receptor agonists increase the activity of serotonergic neurons in the dorsal raphe nucleus (Bermack and Debonnel, 2001). Furthermore, when DTG or SA-4503 (two Sigma-1 receptor agonists) was combined with 8-OH-DPAT, a 5-HT1A receptor agonist, there was an enhanced antidepressant action in rats (Skuza and Rogoz, 2007).

In addition, Sigma-1 receptor modulates the dopaminergic neurotransmission as well. Sigma-1 receptors and dopaminergic neurotransmission are strongly related. The application of Sigma-1 receptor ligands enhances both the spontaneous and the NMDA-induced neuronal activity of dopaminergic neurons in the rat mesolimbic and nigrostriatal dopaminergic systems (Gronier and Debonnel, 1999). Furthermore, the activation of Sigma-1 receptor increases the concentration of extracellular dopamine in both the hippocampus and the frontal cortex (Meurs et al., 2007; Dhir and Kulkarni, 2008). Similarly, the antidepressant activity of ropinirole which is a D2/D3 dopamine receptor ligand is mediated by Sigma receptors (Dhir and Kulkarni, 2008). All these results indicate that the Sigma receptor ligands have ability to modulate the dopaminergic system in the brain.

It has been found that Sigma-1 receptor agonists also affect glutamatergic neurotransmission. They could potentiate the effect of NMDAR antagonists. For example, SA-4503 enhanced the antidepressant activity of amantadine and memantine (they are all NMDA receptor antagonists) in rat (Skuza and Rogoz, 2002, 2006). In addition, dehydroepiandrosterone (DHES) exerts antidepressant effects in rat prelimbic cortex *via* the enhancement of glutamate release (Dong et al., 2007).

Additionally, Sigma-1 receptor might exhibit antidepressant-like behaviors *via* its modulation on neurogenesis. In the subgranular zone of the dentate granule (DG) of olfactory bulbectomized mice (OBX mice), the application of Dehydroepiandrosterone (DHEA) significantly increases neurogenesis through the activation of the Akt/GSK3β-catenin pathway (Moriguchi et al., 2013). Also, in Sigma-1 receptor knockout mice, neurogenesis in DG was significantly impaired, which was proposed to contribute to its depressive behaviors (Sha et al., 2013).

MDD and PD are closely related. As mentioned above, depression is an early non-motor symptom in PD; it appears before the onset of motor symptoms (Poewe et al., 2017). Interestingly, depression is also a risk factor for PD. Patients with depression have high risk of developing PD when they grow old (Frisina et al., 2008). In addition, hypothalamic–pituitary–adrenal (HPA) axis which is involved in depression plays an important role in the development of PD (Du and Pang, 2015). An antidepressant, fluvoxamine maleate which is used to treat MDD, also showed benefits for PD treatment (Dalle et al., 2017). Since Sigma-1 receptor is involved in both PD and MDD, whether it is the link between these two diseases needs further clarification.

## Concluding Remarks

Sigma receptors have been involved in many physiological and pathological processes. They are directly associated with GPCRs, ion channels, and other proteins, acting as a scaffolding protein. Although the important roles of Sigma receptors in PD and MDD have been well revealed, several questions still remain, including: (1) What is the structural basis for Sigma-1 receptors to bind their diverse ligands? (2) Since Sigma-1 receptor could form a range of different types of oligomers, what are the roles of these different oligomers? In addition, Sigma-1 receptor agonists appear to favor low oligomeric states, while its antagonists favor high oligomeric states. Do different agonists and antagonists of Sigma-1 receptor induce distinct cellular effects through these distinct oligomeric states? (3) Sigma receptors interact with so many proteins. How do we design the drug to target the specific disease-related proteins without affecting other interacting proteins? (4) What is the molecular identity of Sigma-2 receptor? (5) The role of the Sigma-2 receptor is still poorly understood, future studies are needed. (6) Although many current medications in the market for MDD show significant affinity for Sigma-1 receptors, whether they act through Sigma-1 receptors in humans remains unanswered. (7) Although Sigma receptors have been demonstrated to be involved in PD and MDD, the mechanisms how dysfunctional Sigma receptors contribute to these diseases require further study.

## Author Contributions

KY reviewed the literature, drafted and revised the manuscript. CW revised the manuscript. TS proposed the topic of the manuscript and revised the manuscript. All authors read and approved the final version.

### Conflict of Interest Statement

The authors declare that the research was conducted in the absence of any commercial or financial relationships that could be construed as a potential conflict of interest.
